# Energy Cost of Standing in a Multi-Ethnic Cohort: Are Energy-Savers a Minority or the Majority?

**DOI:** 10.1371/journal.pone.0169478

**Published:** 2017-01-05

**Authors:** Cathríona R. Monnard, Jennifer L. Miles-Chan

**Affiliations:** Department of Medicine / Physiology, University of Fribourg, Chemin du Musée 5, Fribourg, Switzerland; Weill Cornell Medical College in Qatar, QATAR

## Abstract

**Background:**

The disease risks associated with sedentary behavior are now firmly established, and consequently there is much interest in methods of increasing low-intensity physical activity. In this context, it is a widely held belief that altering posture allocation can modify energy expenditure (EE) to impact upon body weight regulation and health. However, we recently showed the existence of two distinct phenotypes pertaining to the energy cost of standing–with the majority of a Caucasian cohort showing no sustained increase in EE during standing relative to sitting. Here we investigated whether this phenomenon is also observed across a multi-ethnic male cohort.

**Objective:**

To determine the magnitude and time-course of changes in EE and respiratory quotient (RQ) during steady-state standing *versus* sitting, and to explore inter-individual variability in these responses across 4 ethnic groups (European, Indian, Chinese, African)

**Design:**

Min-by-min monitoring using posture-adapted ventilated-hood indirect calorimetry was conducted in 35 healthy, men (20–43 years) during 10 min of steady-state standing *versus* sitting comfortably.

**Results:**

69% of subjects showed little or no increase (<5%) in EE during standing compared to sitting (energy savers). Furthermore, the proportion of energy savers did not significantly differ between ethnic groups, despite ethnic differences in anthropometry; with body weight as the primary predictor of the energy cost of standing maintenance (r^2^ = 0.30, p = 0.001).

**Conclusion:**

Our results indicate that the majority of individuals in a multi-ethnic cohort display a postural energy-saver phenotype. The mechanisms by which the large majority of individuals appear to maintain sitting and standing postures at the same energetic cost remains to be elucidated but is of considerable importance to our understanding of the spontaneous physical activity compartment of EE and its potential as a target for weight regulation.

## Introduction

With growing awareness of the health-related dangers of a modern, sedentary lifestyle there has been increasing interest in methods to increase levels of physical activity. Of particular interest is the low-intensity zone of physical activity, which offers a number of advantages over more traditional, moderate-to-vigorous exercise (i.e., fewer limitations due to individual ability [1] or environmental constraint [2], greater compliance [3–5]). Within this zone, increasing the allocation of posture to standing rather than sitting is perhaps the most simplistic approach aimed at increasing activity levels. However, investigations of the energy cost of standing versus that of sitting have been equivocal–with reported mean increase in energy expenditure (EE) between the two postures ranging from <1 to >100% [6–8]. In addition to methodological differences, such large discrepancies in study findings may be due to the large inter-individual variability in the energy cost of posture maintenance. Indeed, we have recently demonstrated the existence of distinct phenotypes pertaining to the energy cost of standing–with the majority of a Caucasian cohort showing no sustained increase in EE during standing relative to sitting [9]. However, it remains to be determined whether or not this heterogeneity can also be observed across other ethnic groups.

The observation that individuals may show markedly different EE responses to the same challenge is supported by a number of research papers investigating the energetic cost of standardized activities amongst developing and subsistence-level populations [10–14]. In general, these energetic costs are often lower than those observed in more developed countries [12,15,16]. However, such observations are difficult to interpret given large differences in environmental and methodological parameters. Furthermore, comparisons of the energy cost of low-intensity physical activities, such as standing, across ethnic groups, under the same conditions and experimental protocol, are limited [11–14], and do not explore inter-individual variability.

Therefore, the aim of the present study was to determine whether or not the overall energy cost of standing versus sitting is comparable, and if the heterogeneity in response persists, when comparing across four ethnic subgroups (European, Chinese, Indian, and African) under identical, well-standardised measurement conditions. Furthermore, given the influence of body size, proportions and composition on EE, the ethnic-specific differences in these variables [17–19], and their reported association with balance and postural sway [20,21], the present study also sought to investigate the relationship between anthropometry and the energy cost of standing versus sitting in a multi-ethnic group.

## Materials and Methods

### Subjects

Thirty five young, healthy men participated in the present study, with a mean (± SEM) age of 27 ± 1 y, weight of 73 ± 2 kg, and body mass index (BMI; in kg/m^2^) of 23 ± 1. Subjects were either of European (n = 9), Chinese (mainland China; n = 7), Indian (n = 10), or African (Sub-Saharan; n = 9) origin, and currently living in Switzerland. All subjects were weight-stable, with less than 3% body weight variation in the six months preceding the study. Smokers, claustrophobic individuals, individuals taking medication, and those with any metabolic disease were excluded from participating in this study. This sample size was derived using an online power calculator (http://www.statisticalsolutions.net/pssTtest_calc.php), using a one-sided test (as it was expected that any change in EE between sitting and standing would be in the positive direction) based on the data obtained from our previous study [9], an error probably (α) of 0.05 and power (1-β) of 0.80, which indicated a necessary sample size of 6 subjects per group. The study complied with the Declaration of Helsinki and was approved by the Fribourg cantonal ethical committee; all subjects gave written consent.

### Experimental design

Prior to the day of testing, subjects visited the laboratory in order to complete a questionnaire regarding their lifestyle and medical history, and to familiarize themselves with the experimental procedure and equipment. All subjects were requested to avoid physical activity, caffeine, and dietary supplements in the 24h prior to testing. On the day of testing, subjects arrived at the laboratory at 8h00 following a 12h overnight fast. After the subject voided their bladder, body weight, height and sitting height were measured using a mechanical column scale with integrated stadiometer (Seca model 709, Hamburg, Germany). Leg length was calculated as the difference between height and sitting height, with relative leg length subsequently determined as the ratio of leg length to height. Waist (at the umbilicus), mid-thigh, calf, and mid-arm circumferences were measured to the nearest 1 mm using a non-stretch measuring tape according to the methodology of Lohman et al [22]. EE and respiratory quotient (RQ) were measured using the Deltatrac II ventilated hood system (Datex-Ohmeda, Instrumentarium Corp, Helsinki, Finland) adapted for measurement in a variety of postures. As previously described [9], subjects were seated comfortably in a car seat adapted for calorimetric monitoring, with metabolic measurement conducted until stabilization of EE for at least 15 min, after half an hour of rest. During this period, the subject was instructed to relax and avoid movements. The ventilated hood was then removed and the subject asked to stand relaxed and avoiding movements in front of a wooden frame (supported by a metal base) to which the hood was fixed in a vertical position [9]. This postural transition took a maximum of 2 min, with the measurements of EE and RQ during the transition being excluded from the analysis. A preliminary study showed this to be the length of time required to remove the hood, the subject to change posture, the hood to be replaced, the measurements of EE to equilibrate and the heart rate to stabilize. After transition, EE and RQ were recorded for 10 min. Following a second transition period, measurements were continued during a further sitting period lasting at least 15 min. In order to reduce boredom and accompanying stress, subjects were permitted to watch a calm movie or a documentary throughout the metabolic measurements.

### Data and statistical analysis

All data are presented as Mean ± SEM unless otherwise stated. EE phenotype was defined as before [9], using the following criteria:

1. *Energy saver*: Those who showed little or no change in EE (a rise in EE of <5%) during 10 min standing period relative to sitting.
2. *Energy spender*: Those who *i)* increased EE (a rise in EE of >5%) during first 5 min of the 10 min standing period relative to sitting, and *ii)* maintained an elevated EE throughout the entire 10 min standing period (drop in EE during second 5 min <30% of the rise in EE during first 5 of standing period).

The 5% cut-off point used in these criteria is based on data from a previous study [9], which found that energy savers had a significantly lower mean rise in EE than the energy spenders (EE rise <5% vs 11.5%, respectively; p<0.001). Furthermore, as the measurement error of the Deltatrac II indirect calorimeter in terms of VO_2_ and VCO_2_ has been shown to be <3% [23,24], and within-subject coefficient of variability in resting EE measured within our lab is typically around 2% (with a standard deviation of 0.1 kJ/min), this cut-off value also well exceeds that which can be attributed to measurement error and intra-individual variability.

The statistical treatment of data, by Kruskal-Wallis one-way non-parametric ANOVA followed by all-pairwise comparisons, Pearson correlation, Fisher’s exact test, or linear regression, was performed using the computer software STATISTIX 8 (Analytical Software, St. Paul, Minnesota, USA).

## Results

### Energy expenditure (EE) phenotyping

When all subjects were pooled, EE was higher during standing relative to sitting (4.60 ± 0.12 vs 4.31 ± 0.09 kJ/min; p<0.001). The average increase in EE integrated across the 10 min standing period was +6.5 ± 1.1% (0.28 ± 0.04 kJ/min), but a large range of responses was observed: -2.9% to +21.8% (-0.8 to 0.74 kJ/min).

**Fig 1:**
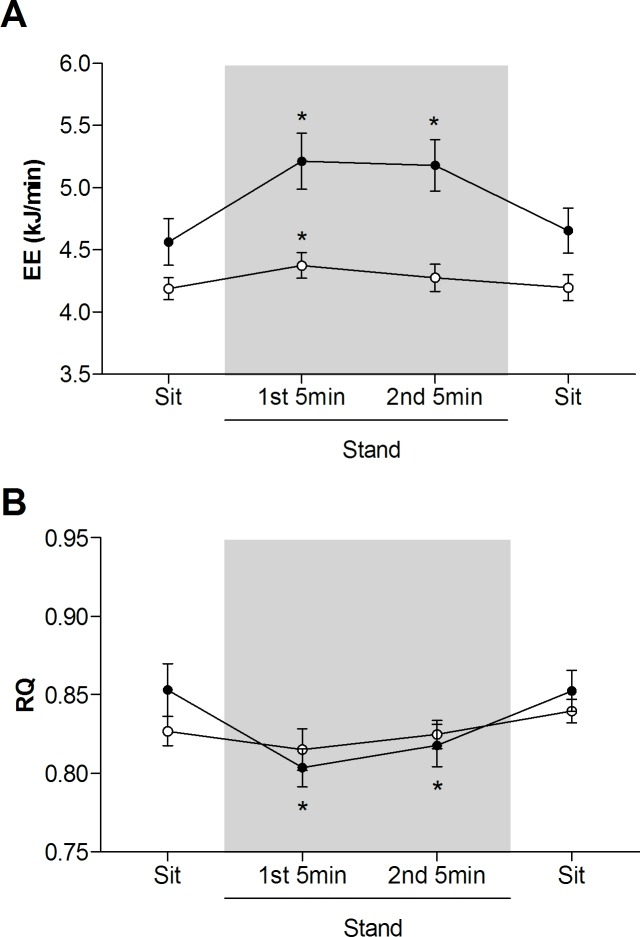
**Energy expenditure (EE; Panel A) and respiratory quotient (RQ; Panel B) responses to standing compared to sitting (subjects grouped by EE response phenotype).** Values mean ± SEM. Open circles represent energy savers; closed circles represent energy spenders. Shaded area indicates 10 min standing period. *statistically significant difference from first (baseline) sitting value.

**Fig 2:**
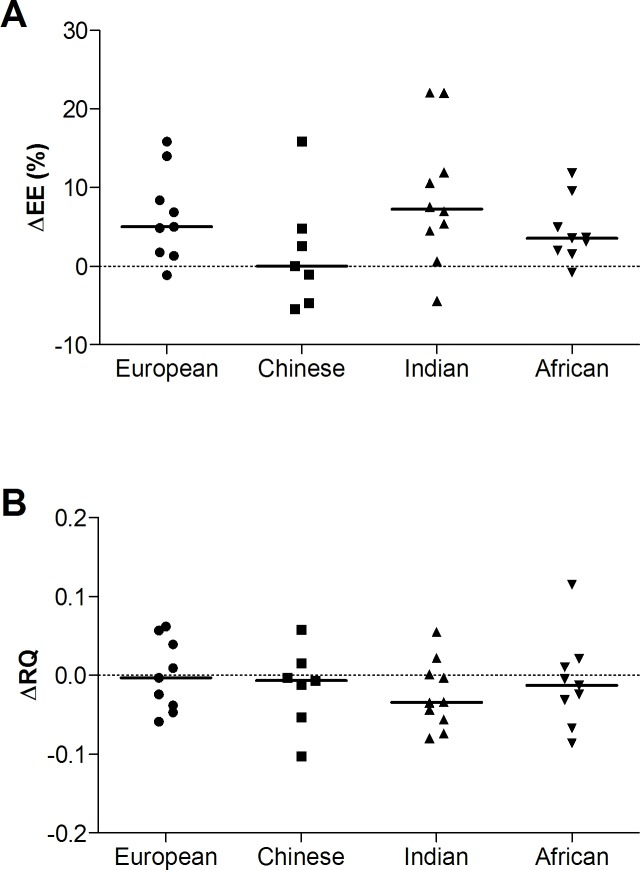
**Individual energy expenditure (EE; Panel A) and respiratory quotient (RQ; Panel B) responses to standing compared to sitting (subjects grouped by ethnicity).** ΔEE is calculated as the change in EE during the second 5 min of the standing period compared to sitting and expressed as a percentage of resting EE. Solid line represents the median value for each ethnic group.

According to the EE response to standing, the majority of subjects (n = 24; 69%) were energy savers, with only 31% (n = 11) showing a sustained increase in EE (on average, 0.63 ± 0.06 kJ/min; 13.9 ± 1.3%) during the 10 min standing period relative to sitting (i.e., energy spenders). The EE and RQ responses of both the energy savers and energy spenders are summarized in **Fig 1**. On average, energy savers had a lower resting EE than energy spenders (4.19 ± 0.10 vs 4.56 ± 0.15 kJ/min; p<0.05), but did not differ significantly in terms of resting RQ (0.827 ± 0.010 vs 0.853 ± 0.015). Similarly, regardless of the response during standing, there were no differences in EE (4.31 ± 0.09 kJ/min vs. 4.34 ± 0.10 kJ/min) or RQ (0.835 ± 0.008 vs 0.843 ± 0.007) between the first (baseline) and second (post-standing) sitting periods, respectively. The distribution of energy savers to energy spenders is shown in **Table 1**, and did not significantly differ between the four ethnic groups (p = 0.4). Individual EE and RQ responses (grouped by ethnicity) are shown in **Fig 2**.

**Table 1:** Distribution of subjects according to energy expenditure (EE) phenotype and ethnicity.

Ethnicity	Energy Savers^1^		Energy Spenders^2^	
	n	*%*	n	*%*
**All**	**24**	***69***	**11**	***31***
European	6	*67*	3	*33*
Chinese	6	*86*	1	*14*
Indian	5	*50*	5	*50*
African	7	*88*	2	*22*

^1^*Energy saver*: Those who showed little or no change in EE (a rise in EE of <5%) during 10 min standing period relative to sitting.

^2^*Energy spender*: Those who i) increased EE (a rise in EE of >5%) during first 5 min of the 10 min standing period relative to sitting, and ii) maintained an elevated EE throughout the entire 10 min standing period (drop in EE during second 5 min <30% of the rise in EE during first 5 of standing period).

Overall, when all subjects were pooled, RQ decreased during standing relative to sitting (0.835 ± 0.008 vs 0.817 ± 0.007; p<0.01), with energy spenders experiencing a significantly greater decrease in RQ during the standing period than energy savers (-0.042 ± 0.007 vs -0.006 ± 0.007; p<0.01) (Fig 1B). However, no significant correlations were observed between EE and RQ at any point during the experimental protocol, either in terms of raw values or change from baseline.

### Anthropometric comparison between ethnic groups

The characteristics of all subjects are summarized in **Table 2**. There were no significant differences in age, height, weight, BMI or waist circumference between the ethnic groups. However, there were differences between groups in terms of body proportions. Most notably, in the Chinese group sitting height was significantly greater than the African group (90.7 + 0.4 cm vs. 85.6 + 1.1 cm, respectively), leg length significantly less than both the European and Indian groups (82.0 + 1.2 vs. 88.9 + 1.6 and 88.6 + 1.0 cm, respectively), and relative leg length (leg length/height) significantly less than both Indian and African groups (0.47 + 0.004 vs 0.50 + 0.004 and 0.51 + 0.004 cm, respectively). Overall, among the 4 ethnic groups studied here, the Chinese group showed the lowest values for body weight, height, leg length, and relative leg length, as well as for arm and waist circumferences.

**Table 2:** Subject characteristics.

	All	European	Chinese	Indian	African	*p*-value
* *	n = 35	n = 9	n = 7	n = 10	n = 9	
**Age**	27.4 ± 0.7	24.9 ± 1.1	27.3 ± 0.6	27.3 ± 1.0	30.2 ± 1.9	NS
(y)	*(20–43)*	*(20–30)*	*(26–30)*	*(21–32)*	*(25–43)*	
**Weight**	73.1 ± 2.4	75.4 ± 4.9	66.9 ± 5.6	73.4 ± 4.4	75.1 ± 5.1	NS
(kg)	*(45–106)*	*(54–106)*	*(45–88)*	*(52–103)*	*(56–106)*	
**Height**	176 ± 1.0	180 ± 2.1	173 ± 1.3	176 ± 1.9	174 ± 2.0	NS
(cm)	*(166–190)*	*(170–190)*	*(168–178)*	*(168–186)*	*(166–183)*	
**BMI**	23.2 ± 0.7	21.9± 1.3	22.3 ± 1.7	23.7 ± 1.3	24.7 ± 1.3	NS
(kg/m^2^)	*(15*.*5–32*.*0)*	*(17*.*5–29*.*4)*	*(15*.*5–29*.*2)*	*(16*.*9–31*.*4)*	*(19*.*1–32*.*0)*	
**Sitting height**	88.4 ± 0.7	90.6 ± 1.2^a,b^	90.7 ± 0.4^a^	87.5 ± 1.4^a,b^	85.6 ± 1.1^b^	<0.05
(cm)	*(80–96)*	*(85–96)*	*(90–92)*	*(83–94)*	*(80–90)*	
**Leg length**	87.3 ± 0.7	88.9 ± 1.2^a^	82.0 ± 1.2^b^	88.6 ± 1.0^a^	88.4 ± 1.3^a,b^	<0.05
(cm)	*(77–94)*	*(85–94)*	*(77–86)*	*(85–94)*	*(82–94)*	
**Relative leg**	0.50 ± 0.002	0.50 ± 0.003^a,b^	0.47 ± 0.004^b^	0.50 ± 0.004^a^	0.51 ± 0.004^a^	<0.001
**length**	*(0*.*46–0*.*53)*	*(0*.*49–0*.*51)*	*(0*.*46–0*.*49)*	*(0*.*49–0*.*52)*	*(0*.*49–0*.*53)*	
**Mid-arm**	30.2 ± 0.7	29.4 ± 0.9^a,b^	27.0 ± 1.4^a^	30.7 ± 1.4^a,b^	33.1 ± 1.4^b^	<0.05
**Circumference (cm)**	*(21–42)*	*(25–34)*	*(21–31)*	*(68–104)*	*(29–42)*	
**Waist**	85.4 ± 1.8	85.8 ± 3.5	82.9 ± 4.6	88.6 ± 3.1	83.6 ± 4.1	NS
**Circumference** (cm)	*(64–105)*	*(73–105)*	*(64–99)*	*(68–104)*	*(66–105)*	
**Mid-thigh**	52.0 ± 1.2	52.9 ± 1.8	50.4 ± 2.7	50.7 ± 2.1	53.9 ± 2.8	NS
**Circumference** (cm)	*(38–68)*	*(43–60)*	*(40–61)*	*(38–63)*	*(43–68)*	
**Calf**	36.7 ± 0.6	37.8 ± 1.2	37.9 ± 1.5	35.2 ± 1.1	36.4 ± 1.2	NS
**Circumference** (cm)	*(31–45)*	*(33–45)*	*(32–43)*	*(32–44)*	*(31–43)*	

Data presented as mean ± SEM *(range)*. NS: not significant. Values not sharing superscript (i.e., a,b) are significantly different (p<0.05) from one another by Kruskal-Wallis test.

### Relationship between EE phenotype and anthropometry

Energy spenders had significantly greater average body weight (82.3 ± 4.0 vs 68.8 ± 2.7 kg; p<0.01), waist circumference (91.6 ± 3.1 vs 82.6 ± 2.1 cm; p<0.05) and leg length (89.8 ± 1.2 vs 86.2 ± 0.8 cm; p<0.05) compared to energy savers.

**Fig 3:**
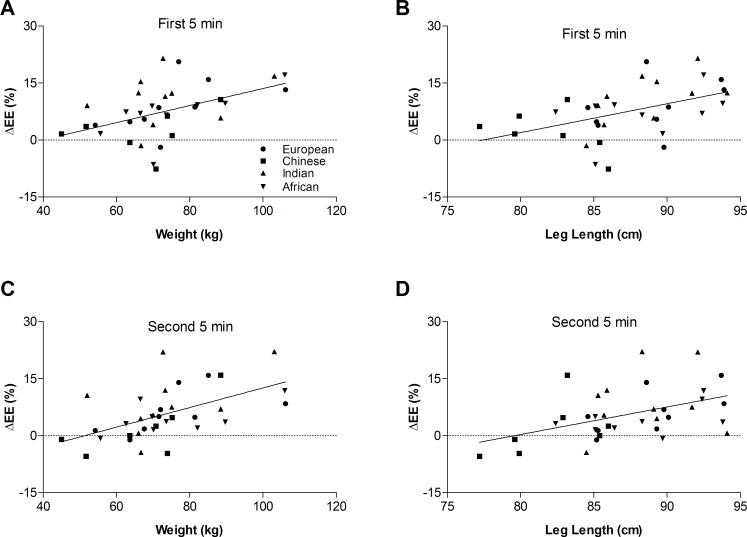
Relationship between the energy cost of standing relative to sitting and body weight (left panels), and leg length (right panels). *Panels A and B* show the relationship with (*A*) the percentage change in EE (ΔEE) and body weight and (*B*) ΔEE and leg length during the *first 5 min* of the standing period compared to resting EE; *Panels C and D* show the relationship with (*C*) ΔEE and body weight and (*D*) ΔEE and leg length during the *second 5 min* of the standing period compared to resting EE. Symbols denote ethnic group (defined in *Panel A*). Solid line indicates the linear regression including all subjects.

Results of the Pearson correlation analyses between change in EE during the first and second 5 min standing periods compared to sitting and the various anthropometric variables are shown in **Table 3**; Significant, positive correlations were observed between the energy cost of standing during the *first 5 min* of the standing period and body weight (Fig 3A) and leg length (Fig 3B) (r^2^ = 0.22, both). Significant, positive correlations were also observed between the energy cost of standing during the first 5 min of the standing period and height, mid-arm- and waist circumferences (r^2^ = 0.11–0.22; data shown in Table 3).

**Table 3:** Correlations (Pearson) between the energy cost of standing posture maintenance and various anthropometric measurements.

* *	%change in energy expenditure			
	(from sitting)			
	First 5 min		Second 5 min	
* *	*r*	*p*	*r*	*p*
**Age**	-0.089	0.612	-0.104	0.554
**Weight**	0.467	**0.005**	0.548	**0.001**
**Height**	0.364	**0.032**	0.407	**0.015**
**BMI**	0.308	0.072	0.380	**0.024**
**Sitting height**	0.046	0.795	0.125	0.474
**Leg length**	0.472	**0.004**	0.460	**0.006**
**Relative leg length**	0.326	0.056	0.270	0.117
**Mid-arm Circumference**	0.361	**0.033**	0.458	**0.006**
**Waist Circumference**	0.426	**0.011**	0.496	**0.002**
**Mid-thigh Circumference**	0.247	0.153	0.356	**0.036**
**Calf Circumference**	0.224	0.195	0.333	0.051

Values highlighted in bold represent statistically significant correlations.

Significant, positive correlations were also observed between the energy cost of standing maintenance (i.e., during the *second 5 min* of the standing period) and body weight (Fig 3C) and leg length (Fig 3D) (r^2^ = 0.30 and 0.21, respectively). During this time-period (standing maintenance), significant, positive correlations were also observed between the energy cost of standing maintenance and height, BMI, circumferences of the mid-arm, waist, and mid-thigh (r^2^ = 0.13–0.30; data shown in Table 3).

These correlated anthropometric variables were then included in stepwise linear regression analyses in addition to age and ethnicity to eliminate auto-correlates. Following the application of these analyses, only leg length remained significant during the first 5 min of standing (r^2^ = 0.22, p<0.005), and only weight remained significant during the second 5 min (r^2^ = 0.30, p = 0.001).

## Discussion

The majority of individuals show little or no increase in energy expenditure during standing relative to sitting, with results of the present study showing the predominance of the postural energy saver phenotype regardless of ethnicity. Whilst the mechanisms by which the large majority of individuals appear to maintain sitting and standing postures at the same energetic cost remains to be elucidated, this study indicates that this observed heterogeneity may, in part, be related to anthropometric differences. In our previous study, we did not observe any relationship between the energy cost of standing posture maintenance in European subjects and body weight, height or composition, which may have been due to the narrow range of the mono-ethnic study cohort or the fact that the previous study had a smaller sample size than the current study (n = 22 vs. 35). However, in the current study we found body weight to be the most consistent and robust contributor to variability in energy cost; accounting for ~30% of the observed individual variability in energy cost.

Whilst the number of subjects in the present study was too small to make a comprehensive comparison, two previous studies have compared the energy cost of standing compared to sitting between ethnic groups, although the results are somewhat discrepant. The first study, by Geissler and Aldouri [12], compared European, Asian (mixed grouping from Hong Kong, South-East Asia, and the Indian subcontinent), and African men, and reported that the energy cost of standing (compared to lying) was higher in the European subjects (by 10 to 13%). This observed difference, of 10–13%, persisted in a subset of subjects matched by weight, height and BMI, leading the authors to conclude that the ethnic differences existed over and above those due to body size. However, body proportions were not assessed, and the observed differences appear to be primarily due to a large difference in resting metabolic rate between the Europeans and the other two groups. In fact, if considering only the change in EE between sitting and standing, the Asian group showed the lowest delta (8% versus 12% and 16% for the European and African groups, respectively). The second study, by Strickland and Ulijaszek [13], compared British and Nepalese (Gurkha) soldiers and found no differences between these two groups in terms of the energy cost of standing, calculated as the difference between standing and lying EE. Again however, when considering only the difference between sitting and standing EE, the difference in EE observed in the British soldiers was more than twice that of the Gurkhas (0.4 vs 0.9 kJ/min, or +6% vs +13%), but this difference was not discussed. Interestingly, Gurkha soldiers also had, on average, shorter legs and greater leg circumferences (mid-thigh and calf) than their British counterparts. However, body proportions (i.e., the ratio of leg length to height) did not appear to differ between the two groups.

In the present study, we also found an association between leg length and energy cost during the first 5 min of the standing period. This association is most likely reflective of the cost of the postural transition (sitting to standing) rather than the maintenance of the standing posture per se; with the cost of transitioning from a chair of equal height greater in those with longer legs [25]. However, the relationship between weight and the energy cost of maintaining a standing posture, and the association with relative leg length, remains to be explained. The answer may indeed lie within leg fat-free mass (either in terms of absolute content, or ratio to fat mass). However, with controversy regarding the appropriateness of most analytic field techniques to compare across ethnic groups given known ethnic differences in body proportions [26–30], a comparison would require sophisticated techniques (such as magnetic resonance imaging), or ethnic-specific algorithms for each ethnic subgroup, which were not available in the present study.

In the current study, energy spenders had greater body weights than energy savers. This association has clinical implications for obese individuals, particularly the energy spenders, who based on their greater body weight, may benefit more in terms of energy expended when substituting standing for sitting. Recent studies highlight modest EE benefits of standing for obese individuals [31,32]. Future studies should focus on uncovering the mechanisms underlying these specific EE phenotypes.

The current study has a number of caveats, in particular: *i)* Given the small sample size for each ethnic group, it is difficult to determine whether some of the large non-significant differences between the groups reflect actual biological differences or simply data variation. Although similar non-significant differences have been shown in sitting / standing interventions among Europeans [9], future work with greater numbers of subjects in each ethnic group is required to explore this further. *ii)* Females were excluded from the current study in order to remove any confounding effect of female hormones on EE. This represents a limitation of the study as it limits the generalizability of the findings, and therefore future studies should include mixed gender ethnic groups. *iii)* Another limitation of the study is that no measures of movement or weight shifting were performed. Subjects were requested to refrain from moving while under the ventilated hood and were restricted in their movement simply as a result of the ventilated hood being securely fastened around them. The influence of movement on the results was further minimized by excluding the two minutes of data associated with the transition from sitting to standing and vice versa; however, it is possible that some individuals performed more micro-movements during standing than others, or more weight-shifting (shifting of body weight from one leg to the other) and that this variability in small movements underlies the differences in energy cost observed. As such, measurement and investigation of these movements merits future investigation, particularly in light of previously reported associations between anthropometry, balance and postural sway [20,21]. Strengths of the current study include the fact that EE was measured minute-by-minute by indirect calorimetry allowing us to determine the EE cost of maintaining a standing posture in a multi-ethnic group and combined with standardized measurements of anthropometry and body composition. The EE cost of posture maintenance was correlated with body weight, such that those classified as having the energy spender phenotype had greater average body weights. This has practical implications for standing interventions aimed at increasing EE in obese individuals. Additionally, identifying EE phenotypes (energy spenders and energy savers) provides a greater understanding of the spontaneous physical activity component of EE regulation. This study also identified a novel association between leg length and the EE cost of posture maintenance, which warrants further exploration.

In conclusion, our results indicate that the majority of individuals in a multi-ethnic cohort display a postural energy-saver phenotype. Those who displayed an energy spender phenotype had a significantly greater average body weight, which has important practical implications for interventions designed to increase EE in the obese. With considerable importance to our understanding of the spontaneous physical activity compartment of EE and its potential as a target for weight regulation, the mechanisms by which the large majority of individuals appear to maintain sitting and standing postures at the same energetic cost warrant further investigation

## Supporting Information

S1 Table Subject characteristics. Individual demographic, anthropometric, and body composition data for each study participant.
(PDF)

S2 Table Individual energy expenditure and respiratory quotient responses to standing compared to sitting. (PDF)
