# Supplementary material for: Energy Cost of Standing in a Multi-Ethnic Cohort: Are Energy-Savers a Minority or the Majority?
Source: PLoS One. 2017 Jan 5;12(1):e0169478. doi: 10.1371/journal.pone.0169478 (PMC5215931; doi:10.1371/journal.pone.0169478)
Supplement: S1 Table — Individual demographic, anthropometric, and body composition data for each study participant. (PDF) [file pone.0169478.s001.pdf]

S1 Table: Subject characteristics. Individual demographic, anthropometric and body composition data for each study participant.

Subject	Ethnicity	Group	Age (y)	Weight (kg)	Height (cm)	BMI (kg/m2)	Sitting Height (cm)	Leg Length (cm)	Relative Leg Length	Mid-Arm Circumference (cm)	Waist Circumference (cm)	Mid-Thigh Circumference (cm)	Calf Circumference (cm)
18	Chinese	Saver	28	45.0	170.5	15.5	90.9	79.6	0.467	20.6	64.0	44.8	32.0
25	Chinese	Saver	27	51.7	167.5	18.4	90.3	77.2	0.461	24.5	71.1	48.0	33.5
27	Chinese	Saver	26	75.2	172.4	25.3	89.5	82.9	0.481	30.0	91.2	57.0	41.0
50	Chinese	Saver	30	63.6	175.0	20.8	89.6	85.4	0.488	25.9	80.3	40.0	37.2
32	Indian	Saver	29	66.7	167.9	23.7	83.4	84.5	0.503	29.4	91.2	54.5	36.5
51	Indian	Saver	32	70.0	171.3	23.9	85.6	85.7	0.500	33.0	85.7	50.7	35.0
28	African	Saver	36	70.1	170.1	24.2	85.0	85.1	0.500	32.5	85.0	56.9	36.0
31	African	Saver	29	55.6	170.5	19.1	80.8	89.7	0.526	28.5	66.4	42.6	31.0
14	African	Spender	29	66.5	177.4	21.1	85.0	92.4	0.521	29.9	74.1	52.6	33.0
29	African	Saver	29	62.6	168.0	22.2	85.6	82.4	0.490	30.4	70.2	52.3	34.7
48	African	Saver	25	73.6	174.5	24.2	86.2	88.3	0.506	29.0	85.0	42.5	39.0
15	European	Saver	22	72.0	183.8	21.3	94.0	89.8	0.489	29.0	80.0	52.2	35.7
42	European	Saver	22	54.2	174.0	17.9	88.7	85.3	0.490	25.0	72.5	43.0	33.0
44	European	Saver	30	81.4	179.0	25.4	88.9	90.1	0.503	30.7	93.3	59.7	41.5
52	Chinese	Saver	28	73.9	171.7	25.1	91.8	79.9	0.465	28.7	89.4	52.4	40.5
6	Indian	Saver	25	66.0	185.9	19.1	91.8	94.1	0.506	24.5	81.3	46.5	32.5
49	Indian	Spender	28	73.3	168.5	25.8	82.6	85.9	0.510	32.0	94.3	52.0	35.5
13	African	Saver	25	82.1	174.5	27.0	88.1	86.4	0.495	34.2	96.1	59.5	36.7
1	African	Spender	28	106.0	182.1	32.0	89.6	92.5	0.508	41.6	105.0	68.0	43.0
7	European	Saver	27	63.7	170.4	21.9	85.2	85.2	0.500	27.3	80.4	51.9	35.4
9	European	Spender	29	77.0	181.1	23.5	92.5	88.6	0.489	28.3	86.0	50.7	38.0
16	European	Spender	25	106.2	189.9	29.4	96.0	93.9	0.494	34.3	105.0	60.0	45.0
22	European	Saver	20	67.6	183.5	20.1	94.2	89.3	0.487	27.8	73.5	48.4	36.2
55	Chinese	Saver	26	70.8	177.9	22.4	91.9	86.0	0.483	28.0	85.1	49.9	38.0
57	Indian	Spender	31	72.7	177.6	23.0	85.5	92.1	0.519	29.6	91.2	49.0	34.2
58	African	Saver	43	69.7	165.5	25.4	80.4	85.1	0.514	37.0	83.5	50.0	35.0
59	Indian	Spender	29	75.1	177.2	23.9	85.5	91.7	0.517	29.0	87.0	37.5	32.5
60	Indian	Saver	26	66.6	172.6	22.4	83.3	89.3	0.517	31.0	86.5	50.3	34.0
61	African	Saver	28	89.7	183.1	26.8	89.3	93.8	0.512	34.6	87.0	61.0	39.6
26	Chinese	Spender	26	88.4	174.0	29.2	90.8	83.2	0.478	31.0	99.0	60.6	42.8
11	Indian	Spender	21	103.1	181.3	31.4	93.0	88.3	0.487	39.5	103.5	62.8	44.0
30	Indian	Spender	26	52.0	175.5	16.9	90.2	85.3	0.486	24.5	67.6	46.5	31.8
5	Indian	Saver	26	88.5	183.0	26.4	93.9	89.1	0.487	34.0	98.0	57.0	36.4
43	European	Saver	25	71.6	172.0	20.0	87.4	84.6	0.492	30.8	86.3	52.8	36.8
46	European	Spender	24	85.1	182.6	17.5	88.9	93.7	0.513	31.6	95.0	57.6	38.8
