# Supplementary material for: Energy Cost of Standing in a Multi-Ethnic Cohort: Are Energy-Savers a Minority or the Majority?
Source: PLoS One. 2017 Jan 5;12(1):e0169478. doi: 10.1371/journal.pone.0169478 (PMC5215931; doi:10.1371/journal.pone.0169478)
Supplement: S2 Table — (PDF) [file pone.0169478.s002.pdf]

S2 Table: Individual energy expenditure and respiratory quotient responses to standing compared to sitting

	Energy Expenditure (kJ/min)				Respiratory Quotient			
Subject	Sit A	Stand (1st 5min)	Stand (2nd 5 min)	Sit B	Sit A	Stand (1st 5min)	Stand (2nd 5 min)	Sit B
18	3.36	3.41	3.33	3.31	0.805	0.860	0.820	0.856
25	3.61	3.74	3.41	3.67	0.777	0.770	0.770	0.832
27	4.46	4.51	4.67	4.39	0.883	1.010	0.780	0.846
50	4.48	4.45	4.48	4.30	0.807	0.788	0.865	0.827
32	3.48	3.43	3.33	3.43	0.914	0.850	0.870	0.905
51	4.26	4.43	4.49	4.28	0.832	0.768	0.752	0.835
28	4.19	3.92	4.25	3.74	0.907	0.890	0.840	0.854
31	3.83	3.89	3.80	3.79	0.791	0.784	0.812	0.828
14	3.79	4.05	4.15	3.99	0.803	0.780	0.790	0.832
29	3.81	4.09	3.93	4.05	0.825	0.850	0.820	0.816
48	4.12	4.39	4.27	4.36	0.872	0.820	0.848	0.859
15	4.72	4.63	5.04	5.09	0.774	0.830	0.750	0.811
42	4.15	4.31	4.20	4.07	0.783	0.714	0.822	0.808
44	4.36	4.74	4.57	4.50	0.787	0.774	0.844	0.808
52	4.29	4.56	4.09	4.35	0.829	0.806	0.826	0.926
6	4.18	4.70	4.21	4.37	0.838	0.760	0.860	0.812
49	4.61	5.14	5.16	4.69	0.842	0.810	0.808	0.833
13	3.51	3.83	3.58	3.60	0.916	0.810	0.830	0.863
1	4.93	5.77	5.51	4.97	0.811	0.790	0.780	0.858
7	4.02	4.21	3.98	3.88	0.754	0.724	0.816	0.796
9	4.23	5.10	4.82	4.22	0.843	0.800	0.840	0.852
16	5.77	6.53	6.25	5.78	0.943	0.850	0.896	0.899
22	4.98	5.25	5.07	4.84	0.843	0.866	0.784	0.842
55	4.47	4.13	4.59	4.20	0.792	0.758	0.780	0.750
57	4.09	4.97	4.99	4.39	0.833	0.812	0.830	0.823
58	4.04	4.40	4.24	4.14	0.805	0.808	0.920	0.856
59	4.87	5.47	5.23	4.57	0.838	0.754	0.782	0.831
60	4.22	4.87	4.41	4.36	0.861	0.896	0.916	0.892
61	4.78	5.24	4.95	5.50	0.820	0.860	0.830	0.830
26	4.97	5.50	5.76	5.05	0.863	0.820	0.810	0.873
11	4.53	5.29	5.53	5.04	0.974	0.890	0.900	0.954
30	3.56	3.88	3.94	3.58	0.815	0.760	0.780	0.823
5	4.75	5.02	5.08	4.43	0.831	0.820	0.832	0.849
43	4.44	4.82	4.67	4.09	0.797	0.746	0.806	0.848
46	4.86	5.63	5.63	4.92	0.818	0.772	0.780	0.798
